# The Challenges in Conducting Economic Evaluations for Rehabilitation Technologies

**DOI:** 10.46292/sci23-00035S

**Published:** 2023-11-17

**Authors:** Brian Chun-Fai Chan

**Affiliations:** 1 KITE Research Institute, Toronto Rehabilitation Institute, University Health Network, Toronto, Canada;; 2 Institute of Health Policy, Management and Evaluation, University of Toronto, Toronto, Canada

**Keywords:** economic evaluation, health technology assessment, rehabilitation

## Abstract

**Background:**

Health technology assessment (HTA) is an important evidentiary component in the decision-making process for the adoption of new healthcare technologies to the healthcare system. Economic evidence is an important consideration in HTAs. Recent systematic reviews in rehabilitation have shown a limited number of economic evaluations and high levels of uncertainty in the results. It is unclear whether there are challenges related to the field of rehabilitation and the technologies used in rehabilitation that inhibit the development of economic evidence.

**Methods:**

In this study, economic evaluations in rehabilitation were reviewed. This was followed by a summary of the latest evidence on the challenges of conducting HTA for medical devices and the relationship with rehabilitation technologies. Finally, several considerations are suggested to improve the HTA of technologies that target rehabilitation. A literature review of Google Scholar and PubMed was conducted to identify reviews in economic evaluations in rehabilitation. A recent review on the barriers to HTA of medical devices in general was also examined to identify similar concerns with rehabilitation technologies.

**Results:**

The challenges identified include the lack of high-quality studies, the interaction between the technology and the user, the short product life cycle, and estimation of efficacy in technologies with multiple target populations.

**Conclusion:**

Overall, many of the challenges in evaluating medical devices also apply to rehabilitation interventions. Further research and discussion on these issues are necessary to increase the clinical evidence for rehabilitation technologies, strengthen the development of HTAs, and facilitate the use of technologies to improve the health of individuals requiring rehabilitation.

## Introduction

In many countries, the evaluation of health technologies is an important step in the decision-making process for adoption into the healthcare system. Health technology is broadly defined by the World Health Organization as “the application of organized knowledge and skills in the form of devices, medicines, vaccines, procedures and systems developed to solve a health problem and improve quality of lives.”^1^ The evaluation of health technology for decision-making or health technology assessment (HTA) has been defined as “a multidisciplinary process that uses explicit methods to determine the value of a health technology at different points in the technology lifecycle. The purpose is to inform decision-making in order to promote an equitable, efficient, and high-quality health system.”^2^ This definition has been criticized for being too vague, containing unhelpful adjectives, and including specific use and purposes, precluding others.^3^ However, despite the critique, this definition has been generally accepted by the HTA community.^2^ Guidelines for the development of HTA are most commonly found for medicines, vaccines, and devices, suggesting that these types of technologies are the most commonly evaluated.^4^ Several HTAs have been published in the last few years on health technologies that may impact SCI care. These include evaluation of intermittent catheters,^5^ electrical stimulation,^6^ negative pressure wound therapy for pressure injury,^7^ intramuscular diaphragm stimulation,^8^ wheelchairs and accessories,^9^ and motorized walking devices.^10^ These reports were developed by HTA agencies that are commissioned by governments to provide independent evidence to inform healthcare decision-makers.^11^-^14^ Many of these reports facilitated the development of recommendations for public healthcare system funders and administrators.

Barriers to the evaluation of healthcare technologies may introduce delays in access to interventions that improve health. This is problematic as our population continues to age^15^,^16^ and the need for rehabilitation grows.^17^ In 2019, it was estimated that globally 2.41 billion individuals have health conditions that will presently or at a future point benefit from rehabilitation.^17^ This represents one in three individuals worldwide. Thus, overcoming barriers to the evaluation of rehabilitation technologies to facilitate adoption into the healthcare system will shrink the gap of unmet medical need. Economic evaluation is a crucial component of HTA that examines the value for money of new interventions. Specifically, these evaluations examine the cost and consequences of alternative health technologies.^18^ The availability of economic evaluations for health technologies is only a fraction of clinical evidence due to the expertise and complexity required to conduct these studies. As such, there is often much uncertainty in the economic evidence in HTAs. It is clear that as new innovations in rehabilitation care are being developed, there is a greater need for HTA evidence to support the inclusion of these innovations into clinical practice. What is unclear is whether there are systematic barriers in place that impose further challenges to the development of HTA evidence, especially economic evidence, to inform rehabilitation. This issue will be explored through the following three questions. Are there barriers in the economic evidence of rehabilitation technologies? If so, what are the barriers? What can be done to address these barriers?

These questions will be examined in three parts. In the first section, several reviews of economic evaluations for rehabilitation in the peer-reviewed scientific literature and in HTA reports will be summarized to examine limitations to the primary studies identified in these reviews. This is followed in the second section with a review of the challenges of the HTA evaluation of medical devices identified in the peer-reviewed and HTA literature as it relates to economic evaluations. These challenges will be explored from a rehabilitation lens. Finally, recent updates to regulatory processes for health technology approvals will be briefly summarized and followed by recommendations for HTAs in rehabilitation.

## Examples of Economic Evaluation of Rehabilitation Technologies

A quick review using medical subject headings (MeSH) “analyses, cost benefit” and “rehabilitation research” in PubMed and “economic evaluation” and “rehabilitation” in Google Scholar was conducted to identify systematic reviews of economic evaluations conducted in rehabilitation published in the last 5 years. A systematic review published by Flemming and colleagues was selected because this study was inclusive of all services in the field of rehabilitation.^19^ The broad inclusion criteria for adult rehabilitation services included physical, occupational therapy, and general rehabilitation published between 2013 to 2020. In total, 129 publications met the eligibility criteria and were included in the full review. This number translates to almost 19 studies published per year or one and a half papers per month. The total number of studies published per year is relatively sparse given that it represents all economic evaluations for the entire field of rehabilitation. The largest proportion of the economic evaluations examined musculoskeletal conditions (58.0%) followed by cardiorespiratory (17.1%) and multiple system conditions (14.7%). Studies evaluating neurological conditions were the least common (10.1%). Examining the individual studies included in the economic review of rehabilitation, stroke and Parkinson's disease were the most commonly reported neurological conditions followed by dementia and traumatic brain injury. There were no studies in spinal cord injury identified. Overall, studies in lower back pain (LBP) and osteoarthritis (including knee, hand, and hip) had the most economic evidence.^19^ A synthesis of the results of these studies was not provided in the review because the focus was on the quality of the economic evaluations. Instead, several recent economic literature reviews have been conducted for LBP and osteoarthritis and are summarized below.

For LBP, two reviews have been conducted.^20^,^21^ A systematic literature review of economic evaluations of noninvasive and nonpharmacological interventions for LBP identified 33 studies between 2000 and 2015.^20^ Between-study comparisons were challenging given the heterogeneity in the evaluation interventions, setting, and methods. The authors concluded that cognitive behavioral therapy with risk stratification care, information provision, spinal manipulation, acupuncture, and medical yoga may be cost-effective.^20^ In a separate review on the cost-effectiveness of exercise therapy for nonspecific neck pain and LBP, 22 studies were identified up to April 2017.^21^ The results suggest that exercise therapy compared to usual care may be cost-effective for subacute and chronic LBP, but not cost-effective compared to other treatments for neck and LBP. However, the authors recommend additional economic evaluations due to the high level of uncertainty in the results.^21^ In summary, several interventions for LBP including exercise therapy, cognitive behavioral therapy, sharing of information, spine manipulation, acupuncture, and medical yoga appear to be cost-effective based on limited evidence. Additional studies are needed in support of these observations and to evaluate other options for treatment available for LBP.

Two studies have reviewed economic evaluations for rehabilitation with hip and/or knee osteoarthritis. A study by Mazzei and colleagues identified 23 studies.^22^ Studies examining exercise interventions had the largest number of studies (*n* = 12), but there were a variety of comparators including physician-delivered standard care, physiotherapy-delivered standard care, exercise, and education. After reviewing the evidence, the authors concluded that exercise interventions alone or in combination with education or diet were cost-effective compared to physician-led standard care or education.^22^ A separate study by Shahabi and colleagues included 20 economic evaluations in the review.^23^ Their review observed physiotherapy exercises (*n* = 11) and integrated rehabilitation interventions (*n* = 6) to be cost-effective.^23^ To summarize, exercise therapies appear to be cost-effective. However, the results should be interpreted with caution given the variability in treatment delivery and the different comparators used in the different studies.

The two reviews in LBP and two studies in osteoarthritis of the hip and/or knee represent the strongest economic evidence in rehabilitation. Although several interventions were considered cost-effective, there was large variability in the intervention evaluated^20^,^21^,^23^ and recommendations for additional economic evaluations.^21^,^23^ The limited economic evidence may be caused by various reasons, including lack of researchers with the expertise in this topic area or a result of a relatively new field that is rapidly evolving.^16^ There may also be factors specific to rehabilitation technologies that make it more challenging, methodologically, to develop economic evaluations. Most healthcare technologies evaluated broadly fall into three areas: medicines, vaccines, and medical devices. Rehabilitation technologies share more similarities with medical devices than medicines and vaccines. Thus, the challenges in evaluating medical devices and the challenges in evaluating rehabilitation technologies are likely similar.

## Challenges to Conducting HTA for Medical Technologies and Its Connection to Rehabilitation

Ming and colleagues conducted a series of reviews to examine the unique challenges in evaluating the broad spectrum of medical devices.^13^ The researchers identified 26 peer-reviewed published articles that examined the differences and challenges of conducting HTAs for medical devices. Challenges were categorized as limited available clinical evidence, device–user interaction, and short product life cycle and frequent upgrades.

The lack of clinical evidence is one of the most commonly reported challenges to the HTA of medical technologies.^24^ One reason for this limitation is the difficulty in conducting blinded randomized controlled trials (RCTs) for medical devices. Medical devices are visually distinct, and the application of the intervention is hard to mask. This makes the blinding process complex and oftentimes infeasible. In rehabilitation, many of these issues are present in robot-assisted therapies. In a review of robot-assisted interventions for individuals with stroke, 46 studies were identified. Almost two-thirds had adequate allocation concealment, more than four-fifths blinded the assessors, but only 15% blinded participants and clinicians.^25^ In 12 studies of participants with multiple sclerosis, none of the participants or therapists were blinded.^26^ For both these reviews, the authors observed that randomization was achievable in most studies, as was blinding of the individual collecting the study data. However, it was difficult to blind participants and clinicians on whether they were receiving the active rehabilitation treatment or receiving a sham treatment.^25^,^26^ The difficulty in blinding is not limited to robotic assistance technologies but is found in physical therapy trials in general. In a review of physical therapy RCTs, only 8% of studies blinded participants to the intervention they received and a quarter blinded the assessors.^27^ Only 2.5% of studies were reported to be double blinded.^27^ This difficulty in conducting blinded RCTs has been acknowledged as one of the challenges in developing evidence in rehabilitation by the National Center for the Dissemination of Disability Research Task Force on Standards of Evidence and Methods.^28^ Limited high-quality RCTs makes it challenging to develop robust economic evaluations that typically depend on the efficacy outcomes to determine incremental cost-effectiveness of a new intervention.

A challenge facing rehabilitation research that was not acknowledged by Ming and colleagues is the small eligible population for study recruitment.^29^ In a review of neurotherapeutic interventions in spinal cord injuries, it was observed that the largest study site recruitment rates were between 1.21 to 2.42 individuals per center per month.^29^ Similar rates were observed for stroke rehabilitation studies where on average 1.5 participants were recruited per site per month.^30^ From the 512 stroke rehabilitation RCTs identified, only 12% had more than 100 participants. Approximately 34% of individuals screened were randomized into the study.^30^ Slow recruitment may result in trials not achieving the targeted sample size and producing results that are not useful or inaccurate or study termination.^30^ Flemming and colleagues’ review of rehabilitation economic evaluations observed that almost three-quarters of included studies had sample sizes below 300 participants.

Another consideration that was not identified in the study by Ming and colleagues but may be a challenge for the clinical evaluation of rehabilitation technologies is the lack of consensus of treatment parameters. For example, in a recent economic review of physiotherapy for individuals with neurological conditions, the treatment duration ranged from 6 weeks to 1 year and frequency from one session per day to one session per month.^31^ In a separate review of virtual reality therapy for individuals, the total therapy time for the different studies ranged from 180 to 1800 minutes.^32^ With such a large range in therapy times, it is difficult for reviews to draw conclusions on the effectiveness and cost-effectiveness of rehabilitation therapies.^31^ The lack of consensus on treatment parameters along with the impracticality of blinding in clinical trials and small study participant pool all present challenges in developing high-quality evidence for the HTA of rehabilitation technologies.

A second category of challenges reported by Ming and colleagues relates to the interaction between the device and the user. Operators of a medical device may require a period of adjustment as they build their expertise in delivering a technology. This period is also known as the “learning curve.” The implication of this period is that the full benefits of a technology will not be observed until the learning period has been completed.^24^ Several recent literature reviews in surgery have identified a number of studies evaluating the learning curve, including 49 different studies for robot-assisted surgery,^33^ 28 for bariatric surgery,^34^ 15 for hip arthroscopy,^35^ and 14 for spine surgery.^36^ In the field of rehabilitation, learning curve has also been noted as a challenge in introducing new technologies. In a study of therapist's experience with lower limb exoskeletons for gait training rehabilitation, a steep learning curve was one of the common challenges identified by the participants.^37^,^38^ Clinicians have reported that training to operate a lower limb exoskeleton was challenging and “technically demanding.”^37^ The need to learn how to use technologies such as the lower limb exoskeleton also reduces the motivation for clinicians to recommend and administer this intervention to their clients.^39^ Additional investments by the healthcare facilities are also necessary as the lack of qualified therapists was a stated barrier to the use of lower limb exoskeleton.^37^ Where qualified therapists were available, scheduling time for therapy sessions was challenging because of the full therapist case load and the time required for these sessions.^37^

A third challenge in developing HTA for medical technologies is related to the short product life cycle.^40^ Many technologies are only available for a few years until they are replaced by a newer device with small incremental improvements.^40^ In balancing the short product life cycle with timely access to medical interventions that may improve health, some regulatory agencies require the minimal evidence necessary to make an informed decision. For instance, the US Food and Drug Administration (FDA) is responsible for market approval of medical devices in the United States. Most devices are categorized as low risk (class I and II) and only require proof of substantial equivalence to another approved device. Highest risk devices (class III) require a full premarket approval review, including evidence for safety and effectiveness.^41^ In a review of the evidence submitted to the FDA for class III cardiovascular devices receiving premarket approval between 2000 and 2007, 65% submitted a single clinical study.^41^ Twenty-seven percent of studies were randomized and 14% were blinded.^41^ The authors noted that these observations are consistent with previous studies of FDA approval of devices in other therapeutic areas.^41^ Together, the low evidence requirements for market approval and short product life cycle do not facilitate high-quality research. However, even for technologies that provide incremental innovation, it is still important to have clinical evidence. With limited evidence, it is unclear whether all incremental innovations improve health outcomes. In some cases, device innovations could increase patient harm. For instance, in a review of hip and knee replacement implant technologies, Nieuwenhuijse and colleagues observed that five well-known device innovations did not improve patient-reported outcomes or function, and they increased revision occurrence.^33^ Similarly, in a review of knee prosthesis improvements over an 18-year timeframe, five of the 11 updates did not result in better survivorship, and several had higher rates of revision.^34^ Unfortunately, a similar evaluation has not been conducted for other rehabilitation technologies.

The challenges summarized above have a direct impact on the ability to conduct an economic evaluation. These types of studies are often embedded into clinical trials by incorporating important economic questions into the data collected from study participants or are dependent on clinical trial outcomes to extrapolate future costs and consequences. The lack of clinical studies limits the development of economic evaluations. Issues like the learning curve introduce an additional level of uncertainty in the economic assessment of these interventions as a comprehensive analysis needs to consider changes in efficacy over time.^42^,^43^ Finally, the short life cycle of technologies introduces frequent changing costs and outcomes of new and existing technologies that are difficult to predict.^42^ This results in economic evaluations that quickly become obsolete after an update or a brand new technology becomes available. This is by no means a comprehensive list of challenges, but it represents some of the issues commonly experienced in clinical research of medical technologies.

## Steps Forward

Acknowledging the limited evidence for medical device approvals, several regulatory agencies have or are in the process of updating requirements. The European Union has recently updated the regulatory process through Medical Device Regulation 2017/745 and 2017/746.^44^,^45^ The new regulations now require the submission of clinical evidence by the manufacturer of high-risk devices to be publicly available in a database.^46^ Expert panels have also been formed to provide external evaluation of the manufacturer-provided clinical evidence to support the decision-makers.^46^ Health Canada has identified the need for better evidence for medical device approvals. They have taken steps to modernize the guidance on the supporting evidence for higher risk medical devices^47^ and have created a public web portal of the clinical evidence provided to Health Canada by the device manufacturer.^48^ This is a part of a larger action plan to improve the safety and effectiveness of medical devices that enter the Canadian market.^49^ Such progress will hopefully encourage greater research in rehabilitation interventions.

The field of rehabilitation may also benefit from the development of a list of HTA questions that are important for rehabilitation technologies. A similar exercise was conducted recently by Von Huben and colleagues for digital health technologies (DHT) to manage chronic diseases.^50^,^51^ Published evaluation frameworks for DHT were identified through a systematic review. The recommendations from these frameworks were mapped on to the EUNetHTA HTA Core Model version 3, an internationally recognized HTA model of topics and questions to be covered in a comprehensive HTA. Where there were recommendations that were not included in the HTA Core Model, new DHT-specific domains and questions were developed. From this study, a list of HTA questions important to DHT was developed.^50^ With a complete list of DHT-specific HTA questions, a second systematic review was conducted on primary studies in DHT for chronic conditions.^51^ The results from this review highlighted the areas of strengths and weaknesses in HTA-related evidence for current DHT studies. A similar process is recommended for rehabilitation, with a literature review of evaluation frameworks for this population being a first step toward identifying domains of interest and ultimately the development of rehabilitation-specific questions.

The lack of clinical evidence may be addressed by improving the difficulties in clinical trial recruitment. Blight and colleagues reviewed several advances that may support the successful completion of SCI clinical studies, such as improved outcome measures for treatment effects and development of prediction models to improve recruitment of participants likely to benefit from treatment.^29^ The authors also recommended infrastructure improvements, such as additional resources for dedicated research personnel trained to perform neurological and functional assessments, stronger coordination between research centres, and greater involvement from nonprofit nongovernmental organizations.^29^ These advancements and recommendations may facilitate the successful completion of studies resulting in greater availability of evidence and may also support the completion of timely economic evaluations through shorter time to trial completion.

Finally, it is important that decision-makers are aware of the unique characteristics of rehabilitation and the implications for evaluation of technologies that impact this population. Clinicians, researchers, and individuals with lived experience are encouraged to participate in decision-making bodies to ensure that rehabilitation and the technologies that may improve the health of individuals requiring rehabilitation are a part of the conversation. It is important that individuals with direct or indirect experience with rehabilitation are included in healthcare policy deliberations to interpret evidence, form recommendations, or facilitate future study to help shape better healthcare systems for individuals requiring rehabilitation.

The key challenges and recommendations explored in this article are summarized in **Table 1**. There is a need to educate those with a professional or personal interest in rehabilitation on the importance of HTA. Further research and discussion on the enablers and barriers to HTA of rehabilitation technologies is necessary to facilitate the implementation of technologies that can improve the healthcare for individuals requiring rehabilitation.

**Table 1.** Key messages on the challenges of conducting economic evaluation on rehabilitation technologies

**Issue**	**Challenge**	**Barriers**	**Recommendations**
Health technology assessments provide evidence to support funding and integration of new health care technologies for individuals requiring rehabilitation.	There may be barriers to the development of health technology assessments (HTAs) in rehabilitation that may delay or prevent the introduction of technologies to the health care system.	– Lack of clinical evidence (issues with blinding, recruitment, and treatment parameters) – Device and user interaction (learning curve) – Short life cycle (limited requirements for approvals, frequent upgrades)	– Development of rehabilitation specific HTA questions – Improvements in clinical trial methodology and dedicated resources to facilitate completion – Participation of clinicians, researchers, and persons with lived experience in healthcare decision-making bodies
